# Supplementary material for: Detailed anatomy of the braincase of Macelognathus vagans Marsh, 1884 (Archosauria, Crocodylomorpha) using high resolution tomography and new insights on basal crocodylomorph phylogeny
Source: PeerJ. 2017 Jan 19;5:e2801. doi: 10.7717/peerj.2801 (PMC5251941; doi:10.7717/peerj.2801)
Supplement: Supplemental Information 1 — Includes specimen list, character list, additional cladograms depicting the support measures and the synapomorphy list for each node. [file peerj-05-2801-s001.doc]

**Supplementary Information**

**Detailed anatomy of the braincase of *Macelognathus vagans* Marsh, 1884 (Archosauria, Crocodylomorpha) using high resolution tomography and new insights on basal crocodylomorph phylogeny**

Juan M. Leardi^1*^, Diego Pol^2^, and James M. Clark^3^

^1^ Universidad de Buenos Aires, CONICET, Instituto de Estudios Andinos “Don Pablo Groeber” (IDEAN), Facultad de Ciencias Exactas y Naturales, Departamento de Ciencias Geológicas, Intendente Güiraldez 2160, Ciudad Universitaria – Pabellón 2, Buenos Aires, C1428EGBA, Argentina

^2^ Museo Paleontológico Egidio Feruglio, CONICET, Avenida Fontana 140, Trelew 9100, Chubut, Argentina

^3^ Department of Biological Sciences, George Washington University, 2029 G St. NW, Washington, DC, 20052, U.S.A

^*^ Corresponding author: Universidad de Buenos Aires, Instituto de Estudios Andinos “Don Pablo Groeber” (IDEAN),Facultad de Ciencias Exactas y Naturales, Departamento de Ciencias Geológicas, Intendente Güiraldez 2160, Ciudad Universitaria – Pabellón 2, Buenos Aires, C1428EGBA, Argentina, jmleardi@gl.fcen.uba.ar

**Contents**

| **Raw CT data** | 3 |
| --- | --- |
| **Phylogenetic analysis** | 5 |
| Taxon list | 5 |
| Character list | 7 |
| Data matrix | 14 |
| Consensus tree | 19 |
| Nodal support | 19 |
| List of synapomorphies | 24 |
| **Suplementary Literature Cited** | 33 |

**Raw CT Data**

The CT data and the 3D models (STL format) are available upon request to the corresponding author or at the following website http://morphobank.org/permalink/?P2550. However, here we include a Supplementary figure depicting some slices to illustrate the levels of contrast and the quality of the data acquired. The specimen was mounted in a mesh made from foam rubber to protect it during the scanning process. The structure of such mesh can be observed at the background of each slide.


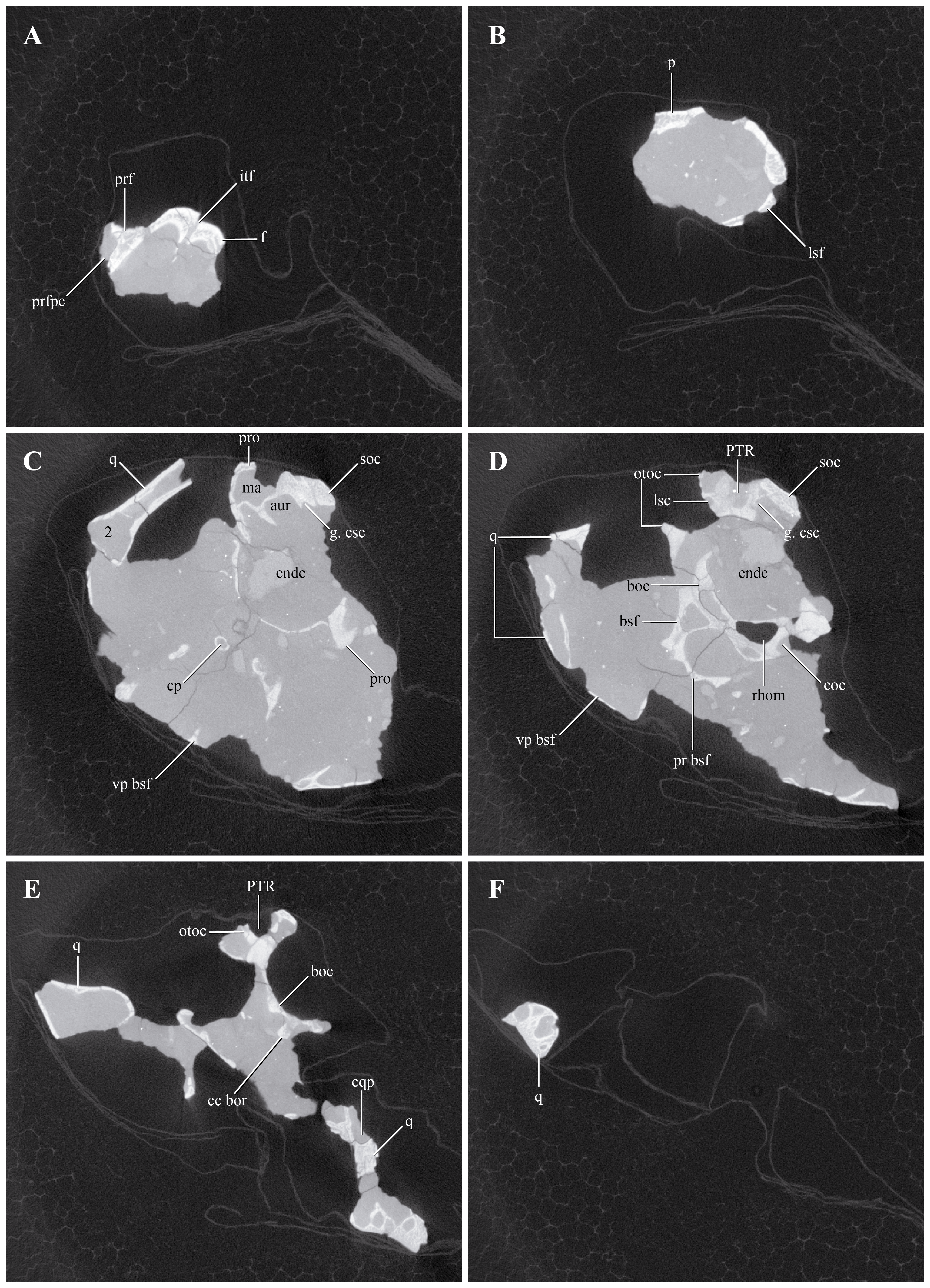


**Figure 1S-** CT slices at the level of: anterior end of the braincase (A); orbits (B); anterior end of the quadrate (C); otic capsule (D); posterior to the otic capsule (E); and, posterior end of the right quadrate (F). Abbreviations are the same as the main manuscript figures, except for: endc, endocranial cavity. Note the highly pneumatic quadrate (C, E,and F).

**Phylogenetic Analysis**

**Taxon list**

Collection numbers of the specimens that were studied first-hand by the authors are added after the bibliographic reference.

*Alligator mississippiensis* (Clark, 1994; Brochu, 1999; FMNH 8201)

*Almadasuchus figarii* (Pol et al., 2013; MPEF PV 3838)

*Baurusuchus albertoi* (Nascimento & Zaher, 2010)

*Baurusuchus salgadoensis* (Carvalho, Campos & Nobre, 2005; MPMA 62-0001-02; UFRJ DG 285-R)

*Cricosaurus araucanensis* (Gasparini & Dellape, 1976; MLP 72-IV-7-1, MLP 72-IV-7-2, MLP 72-IV-7-3, MLP 72-IV-7-4, MLP 72-IV-7-4, MLP 86-XI-5-7, MACN-N 95, MACN-N 64)

*Carnufex carolinensis* (Zanno et al., 2015; Dyrmala & Zanno, 2016)

CM 73372 (Nesbitt, 2011; CM 73372)

*Crocodylus niloticus* (Clark, 1994; Brochu, 1999; FMNH 17157, 217153)

*Dakosaurus andiniensis* (Vignaud and Gasparini, 1996; MHNSR PV 344, MOZ 6146P)

*Dibothrosuchus elpahros* (Wu and Chatterjee, 1993; IVPP V 7907)

*Dromicosuchus grallator* (Sues et al., 2003; NCSM 13733)

*Erpetosuchus granti* (Benton & Walker, 2002)

*Gavialis gangeticus* (Clark, 1994; Brochu, 1999; MLP s/n, FMNH 82681, FMNH 98864)

*Gobiosuchus kielanae* (Osmólska, 1972; Osmólska, Hua & Buffetaut, 1997; ZPAL MgR-II/67, ZPAL MgR-II/68, ZPAL MgRII/69, ZPAL MgR-II/70, ZPAL MgR-II/71)

*Goniopholis simus* (Mook, 1942; Clark, 1986, 1994; Salisbury et al., 1999; NHMUK OR 41098)

*Gracilisuchus stipanicicorum* (Romer, 1972; MCZ 4117, MCZ 4118, PVL 4597, PVL 4612)

*Hallopus victor* (Walker, 1970; YPM 1914)

*Hemiprotosuchus leali* (Bonaparte, 1972; PVL 3829)

*Hesperosuchus agilis* (Colbert, 1952; Clark, Sues & Berman, 2000; AMNH FR 6758, CM 29894)

*Hsisosuchus chowi* (Peng and Zhu, 2005; ZDM 0146)

*Junggarsuchus sloani* (Clark et al., 2004; IVPP V 14010)

*Kayentasuchus walker* (Clark & Sues, 2002; UCMP 131830)

*Litargosuchus leptorhynchus* (Clark & Sues, 2002; BP/1/5237)

*Macelognathus vagans* (Marsh, 1884; Ostrom, 1971; Göhlich et al., 2005; YPM 1415; LACM 5572/150148, LACM 4684/128271, LACM 4684/128272, LACM 4684/128271, LACM 5572/150211, LACM 4684/133772)

*Metriorhynchus superciliosus* (de Blainville, 1853; Wenz, 1968; AMNH 997, SMNS 10116)

*Notosuchus terrestris* (Gasparini, 1971; MLP 64-IV-16-1, MLP 64-IV-16-5, MLP 64-IV-16-6, MLP 64-IV-16-10, MLP 64-IV-16-11, MLP 64-IV-16-12, MLP 64-IV-16-13, MLP 64-IV-16-23, MACN-RN 1037, MACN-RN 1040, MACN-RN 1041, MACN-RN 1042, MACNRN1043, MACN-RN 1044, MUC-PV 147, MUC-PV 209, MUC-PV 287, MUC-PV 900, MPCA-PV 249, MPCA-PV250)

*Orthosuchus stormbergi* (Nash, 1975; SAM-K 409)

*Pelagosaurus typus* (Eudes-Deslongchamps, 1864; NHMUK OR 32599, BSP 1925.I.34, BSP 1990.VIII.68, MB 1925.1, MB R.2883, SMNS 8666, SMNS 80066)

*Postosuchus kirkparticki* (Chaterjee, 1985; Long & Murry 1995; Weinbaum, 2002; Peyer et al., 2008)

*Protosuchus haughtoni* (Busbey and Gow, 1984; BPI/1/4746, BPI/1/4946, BPI/1/4770, BPI/1/5290)

*Protosuchus richardsoni* (Colbert and Mook, 1951; AMNH 3024, MCZ 6727, UCMP 34634, 130860, 131827)

*Pseudhesperosuchus jachaleri* (Bonaparte, 1972; PVL 3830)

*Redondavenator quayensis* (Nesbitt et al., 2005)

*Saltoposuchus conectens* (Sereno & Wild, 1992; SMNS 12352, 12591a, 12596, 12597

*Saurosuchus galilei*

*Sichuanosuchus shuhanensis* (Wu et al., 1997; IVPP V 10594)

*Simosuchus clarki* (Buckley et al., 2000; Krause and Kely, 2010; Georgi and Krause, 2010; Sertich and Groentke, 2010; UA 8679; FMNH PR 2596; FMNH PR 2597; FMNH PR 2598)

*Sphenosuchus acutus* (Walker, 1990; SAM-PK 3014)

*Stagonolepis robertsoni* (Walker, 1961)

*Terrestrisuchus gracilis* (Crush, 1984)

*Trialestes romeri* (Reig, 1963; PVL 2561)

*Zosuchus davidsoni* (Pol and Norell, 2004a; IGM 100/1304, IGM 100/1305, IGM 100/1306, IGM 100/1307, IGM 100/1308)

**Institutional Abbreviations—AMNH**, American Museum of Natural History, New York, USA; **BPI**, Bernard Price Institute, Johannesburg, South Africa; **BSP**, Bayerische Staatssammlung fur Palaontologie und Geologie, Munich, Germany; **FMNH**, Field Museum of Natural History, Chicago, Illinois, USA; **IGM**, Mongolian Institute of Geology, Ulaan Bataar, Mongolia; **IVPP**, Institute of Vertebrate Paleontology and Paleoanthropology, Beijing, China; **LACM**, Natural History Museum of Los Angeles County, Los Angeles, California, USA; **MACN**, Museo Argentino de Ciencias Naturales, Buenos Aires, Argentina; **MB**, Institut fur Palaontologie, Museum fur Naturkunde, Humbolt-Universitat, Berlin, Germany; **MCZ**, Museum of Comparative Zoology, Harvard University, Cambridge, Massachusetts, USA; **MHNSR**, Museo de Historia Natural de San Rafael, San Rafael, Argentina; **MLP**, Museo de La Plata, La Plata, Argentina; **MPCA-PV**, Museo Carlos Ameghino, Cipoletti, Argentina; **MPEF**, Museo Paleontologico Egidio Feruglio, Trelew, Argentina; MPMA, Museu de Paleontologia de Monte Alto, Brazil; **MUC-PV**, Museo de Geologia y Paleontologia, Universidad Nacional del Comahue, Neuquen, Argentina; MOZ, Museo Profesor J. Olsacher, Zapala, Argentina ; **PVL**, Instituto Miguel Lillo, Tucuman, Argentina; **SAM**, Iziko-South African Museum, Cape Town, South Africa; **UA**, University of Antananarivo, Madagascar; **SMNS**, Staatliches Museum fur Naturkunde, Stuttgart, Germany; **UCMP**, University of California Museum of Paleontology, Berkeley, California, USA; **UFRJ**, Universidade Federal do Rio de Janeiro, Departamento de Geologia, Brazil; **YPM**, Peabody Museum of Natural History, Yale University, New Haven, Connecticut, USA; **ZPAL**, Instytut Paleobiologii PAN, Warszawa, Poland.

**Character list**

The character list of the data matrix used in the phylogenetic analysis is supplied bellow. The character list is extended from Pol et al. (2013), which in turn represents an expanded version of Clark et al. (2004). Fifteen characters were considered as ordered (characters 16, 20, 22, 23, 31, 46, 59, 60, 63, 64, 67, 74, 77, 82, 83, and 87) and are also indicated in the list.

1. Posterodorsal process of the premaxilla overlaps anterodorsal surface of the maxilla (0) or dorsal process of premaxilla vertical, strongly sutured to maxilla (1).
2. Facial portion of maxilla anterior to anterior edge of antorbital fenestra equal in length or longer than portion posterior to anterior edge of fenestra (0) or shorter than posterior portion (1).
3. Maxillae do not meet on palate (0) or meet on palate to form secondary bony palate anterior to choana (1).
4. Jugal participates in posterior margin of antorbital fenestra (0) or is excluded by lacrimal or maxilla (1).
5. Descending process of prefrontal absent (0) or present (1).
6. Descending process of prefrontal does not contact palate (0) or contacts palate (1).
7. Prefrontal not underlying anterolateral edge of frontal to a significant degree (0) or with distinct posterior process underlying frontal dorsal to orbit (1).
8. Postfrontal present (0) or absent (1).
9. Dorsal surface of frontal flat (0) or with longitudinal median ridge (1).
10. Squamosal not significantly overhanging lateral temporal region (0) or with broad lateral expansion overhanging lateral temporal region (1).
11. Descending process of squamosal anterior to quadrate present (0) or absent (1).
12. Squamosal without ridge on dorsal surface along edge of supratemporal fossa (0) or with ridge (1).
13. Lateral edge of squamosal without (0) or with longitudinal groove (1).
14. Quadratojugal extending anterodorsally to contact postorbital (0) or not contacting postorbital (1).
15. Quadrate not in contact with prootic (0) or contacting prootic (1).
16. In presumed adults, parietals separate (0), interparietal suture partially obliterated (1), or interparietal suture absent (2). (Ordered).
17. Posteroventral edge of parietals extending more than half the width of the occiput (0) or less than half the width of the occiput (1).
18. Medial margins of supratemporal fossae on lateral surfaces of parietals separated on midline by broad, flat area (0) or by sagittal crest (which may be divided by median sulcus) (1).
19. Occipital margin of parietals V-shaped in dorsal view (0) or straight (1).
20. Exoccipitals broadly separated dorsal to foramen magnum (0), approaching midline without contacting (1), or contacting below supraoccipital (2). (Ordered).
21. Prootic broadly contacting anterior surface of paroccipital process (0) or not in broad contact (1).
22. Depression for mastoid antrum: absent (0), present on lateral surface of prootic dorsal to otic capsule (1), or entering deeply into prootic or connecting with each other through supraoccipital (2). (Ordered).
23. Depression for posterior tympanic recess: absent (0), depression posterior to fenestra ovalis on anterior surface of paroccipital process (1), or penetrating prootic and paroccipital process (2). (Ordered).
24. Paroccipital process dorsoventrally tall and distinctly expanded distally (0) or process narrower dorsoventrally, distal end only slightly expanded (1).
25. Basipterygoid processes of basisphenoid present (0) or absent (1).
26. Basipterygoid processes simple, without large cavity (0) or greatly expanded, with large cavity (1).
27. Articular without dorsomedial projection posterior to glenoid fossa (0) or with dorsomedial projection (1).
28. Posterior edge of maxillary and more posterior dentary teeth concave or straight (0) or distinctly convex (1).
29. Coracoid subcircular in lateral view (0), with elongate, tapering postglenoidal process posteromedially (1), with extremely elongate posteromedial process (2) or with elongate ventromedial process expanded ventrally (3).
30. Proximal ends of metacarpals overlap (0) or abut one another without overlapping (1).
31. Proximal head of femur confluent with shaft (0), or with distinct, medially directed head set off from shaft, representing less than 55% the total width (1), or medially directed head and projected more than 55% the total width (2). (Ordered). [Modified from Clark *et al*. (2004) adding character state 2].
32. Tibia/femur length ratio: less than 1 (0), greater than 1 (1).
33. Anterior edge of paramedian dorsal osteoderms straight (0) or with anterior process (1).
34. Paramedian dorsal osteoderms flat (0) or with distinct longitudinal bend near lateral edge (1).
35. Basisphenoid body similar in size to basioccipital (0) or greatly expanded with pneumatic cavities (1).
36. Exoccipital does not contact distal end of quadrate (0) or contacts quadrate, enclosing internal carotid artery (1).
37. Jugal straight below infratemporal fenestra (0) or strongly arched dorsally (1).
38. Ventral edge of jugal flat or convex (0) or with longitudinal concavity (1).
39. Lateral end of paroccipital process convex (0) or concave (1).
40. Supratemporal portion of postorbital forms squared-off anterolateral corner to rectangular skull roof (0) or postorbital with oblique, anterolaterally facing edge so that skull roof and supratemporal fenestra narrow anteriorly (1).
41. Horizontal shelf in posterior part of supratemporal fenestra poorly developed or absent (0) or well developed, flooring posterior 1/3 of supratemporal fossa (1).
42. Occipital portion of parietal narrow (0) or broad (1).
43. Surangular foramen absent (0) or present (1).
44. *M. pterygoideus posterior* insertion area on angular does not extend onto lateral surface (0) or extends well onto lateral surface (1).
45. Anterior maxillary teeth similar in size to posterior teeth (0) or much larger than posterior teeth (1).
46. Quadrate fenestra: absent (0); present but small (1); or present but large (2). (Ordered). [Modified from Clark *et al*. (2004) adding character state 2].
47. Vertebral centra shallow amphicoelous (0) or procoelous (1).
48. Well-developed hypapophyses absent on cervical vertebrae (0) or present (1).
49. Zygapophyses of anterior dorsal vertebrae sub-vertically oriented (0) or horizontal (1).
50. Osteoderms present (0) or absent (1).
51. Anterior and posterior borders of scapula similar, blade relatively narrow (0) or anterior edge more strongly concave than posterior, blade much broader (1).
52. Glenoid fossa on scapula ventrally or posteroventrally oriented (0) or posterolaterally oriented (1).
53. On distal end of ulna, medial articulation with elongate radiale not confluent with distal articulation with ulnare (0) or articulations confluent, distal end broadly arched (1).
54. First manus digit faces ventrally (0) or faces laterally, flexing towards digit II (1).
55. First metacarpal similar in thickness to second metacarpal or thicker (0) or more slender (1).

Character 56 (Pol *et al*., 2009: Character 125). Posterodorsal process of premaxilla: absent (0), or present extending posteriorly wedging between maxilla and nasals (1).

Character 57 (Clark, 1994: Character 13). Nasal contribution to narial border: yes (0), or no (1).

Character 58 (Clark, 1994: Character 66). External nares: divided by a septum (0), or confluent (1).

Character 59 (modified from Clark, 1994: Character 6). External nares facing anterolaterally or anteriorly (0), dorsally not separated by premaxillary bar from anterior edge of rostrum (1), or dorsally separated by premaxillary bar (2). (Ordered).

Character 60 (modified from Clark, 1994: Character 3). Rostrum proportions: narrow oreinirostral (0), broad oreinirostral (1), nearly tubular (2), or platyrostral (3). (Ordered).

Character 61 (modified from Clark, 1994: Character 79). Maxillary tooth size variation: absent or single wave of size variation (0), or enlarged maxillary teeth curved in two waves (festooned) (1).

Character 62 (Pol *et al*., 2009: Character 139). External surface of maxilla and premaxilla: with a single plane facing laterally (0), or with ventral region facing laterally and dorsal region facing dorsolaterally (1).

Character 63 (Clark, 1994: Character 67). Antorbital fenestra: as large as orbit (0), about half the diameter of the orbit (1), much smaller than the orbit (2), or absent (3). (Ordered).

Character 64 (modified from Clark, 1994: Character 1). External surface of dorsal cranial bones: smooth (0), slightly grooved (1) and heavily ornamented with deep pits and grooves (2). (Ordered).

Character 65 (Clark, 1994: Character 21). Frontals: paired (0), unpaired (1).

Character 66 (Clark, 1994: Character 24). Supratemporal roof dorsal surface: complex (0), or dorsally flat ‘skull table’ developed, with postorbital and squamosal with flat shelves extending laterally beyond quadrate contact (1).

Character 67 (Clark, 1994: Character 37). Palatines: do not meet on palate below the narial passage (0), form palatal shelves that do not meet (1), or meet ventrally to the narial passage, forming part of secondary palate (2). (Ordered).

Character 68 (modified from Clark, 1994: Character 39). Choanal opening: continuous with pterygoid ventral surface except for anterior and anterolateral borders (0), or opens into palate through a deep midline depression (choanal groove) (1).

Character 69 (modified from Clark, 1994: Character 42 by Ortega *et al*., 2000: Character 139). Depression on primary pterygoidean palate posterior to choana: absent or moderate in size being narrower than palatine bar (0), or wider than palatine bar (1).

Character 70 (Clark, 1994: Character 38). Pterygoid: restricted to palate and suspensorium, joints with quadrate and basisphenoid overlapping (0), or pterygoid extends dorsally to contact laterosphenoid and form ventrolateral edge of the trigeminal foramen, strongly sutured to quadrate and laterosphenoid (1).

Character 71 (Clark, 1994: Character 43). Pterygoids: form posterior half of the choanal opening (0), or completely enclose choana (1).

Character 72 (Clark, 1994: Character 41). Pterygoids posterior to choanae: separated (0), or fused (1).

Character 73 (Pol *et al*., 2009: Character 133). Acute anterior process of ectopterygoid projecting along medial surface of jugal: developed (0), or reduced or absent (1).

Character 74 (Pol et al., 2013). Post-temporal fenestra: large and enclosed by parietal, squamosal, and exoccipital, well separated from the supraoccipital (0), or large and enclosed by the squamosal and exoccipital, with its medial end located close to the lateral edge of the supraoccipital (1), or small and with supraoccipital participating from its medial margin (2). (Ordered).

Character 75 (Pol et al., 2013). Subtriangular concavity located on the posterolateral surface of the squamosal, located posteriorly to the otic shelf recess and anterolaterally from the paroccipital process: absent (0), or present (1).

Character 76 (Pol et al., 2013). Squamosal contact with the posterodorsal surface of the quadrate closing posteriorly the otic recess: absent (0), or present (1).

Character 77 (Clark, 1994: Character 49). Quadrate, squamosal, and otoccipital: do not meet to enclose cranioquadrate passage (0), enclose passage near lateral edge of skull (1), or meet broadly lateral to the passage (2). (Ordered).

Character 78 (Pol et al., 2013). Lateral margin of squamosal-postorbital along the upper temporal bar, convex or straight (0), or laterally concave (1).

Character 79 (Ortega *et al*., 2000: Character 75). Anterior opening of temporo-orbital in dorsal view exposed (0), or hidden in dorsal view and overlapped by squamosal rim of supratemporal fossa (1).

Character 80 (Clark, 1994: Character 52). Eustachian tubes: not enclosed between basioccipital and basisphenoid (0), or entirely enclosed (1).

Character 81 (Clark, 1994: Character 56). Basisphenoid: exposed on ventral surface of braincase (0), or virtually excluded from ventral surface by pterygoid and basioccipital (1).

Character 82 (modified from Wu & Sues, 1996: Character 24 and Wu *et al*., 1997: Character 124). Jaw joint: placed at level with basioccipital condyle (0), below basioccipital condyle about level of lower toothrow (1), or below level of toothrow (2). (Ordered).

Character 83 (modified from Clark, 1994: Character 77). Splenial involvement in symphysis in ventral view: not involved (0), involved slightly in symphysis (1), or extensively involved (2). (Ordered).

Character 84 (Ortega *et al*., 1996: Character 9). Ventral exposure of splenials: absent (0), or present (1).

Character 85 (modified from Clark, 1994: Character 71). Retroarticular process: very short and robust projecting ventrally (0), with an extensive rounded, wide, and flat (or slightly concave) surface projected posteroventrally and facing dorsomedially (1), posteriorly elongated, triangular-shaped and facing dorsally (2).

Character 86 (modified from Clark, 1994: Character 83 by Ortega *et al*., 2000: Character 121). Coracoid length: up to two-thirds of the scapular length (0), or subequal in length to scapula (1).

Character 87 (modified from Benton & Clark, 1988: Character e) Radiale: not elongated (0); elongated (1); or greatly elongated, being at least 30% the length of the humerus or femur (2). (Ordered).

Character 88 (modified from Clark, 1994: Character 85). Pubis distal expansion: absent (0), or present, markedly expanded distal end (1).

Character 89 (Clark, 1994: Character 86). Pubis: forms anterior half of ventral edge of acetabulum (0), or pubis partially or completely excluded from the acetabulum by the anterior process of the ischium (1).

Character 90 (Pol et al., 2013). Long axis of the femoral head and axis that joins the fibular and medial condyles at the distal femoral end: forming an angle of 10 degree or more (0), or parallel to each other (1).

Character 91 (Pol et al., 2013). Lesser trochanter (trochanteric crest) in the anterolateral proximal femur: present as a long ridge (0), trochanter absent with/without a distinct scar for muscular attachment (1).

Character 92 (Pol et al., 2013). Pseudointernal trochanter (sensu 36) in the posterolateral proximal femur for insertion of the *M. pubo-ischio-femoralis externus* (PIFE) muscle: absent (0), present (1).

Character 93 (Pol et al., 2013). Development of the fourth trochanter, as a wide knob (0), as a sharp ridge (1).

Character 94 (Pol et al., 2013). Development of distal condyles: fibular condyle at the same level as the medial condyle (0), or fibular condyle slightly distal than the medial condyle (1), or fibular condyle further distally than the medial condyle (2).

Character 95 (modified from Clark, 1994: Character 97 by Ortega *et al*., 2000: chars. 107 and 108). Rows of dorsal osteoderms: two parallel rows (0), more than two rows (1).

Character 96 (Pol et al., 2013). Basisphenoid-exoccipital suture: absent (0), or interdigitated suture lateral to the lateral Eustachian foramina.

Character 97. (NEW) Supraoccipital: fused with the exoccipital (0); or, as a separate ossification (1).

Character 98. (NEW) Supraoccipital shape: narrow, being dorsoventrally taller than lateromedially wide (0); or, wide, being lateromedially wider than dorsoventrally high (1).

Character 99. (modified from Clark, 1986) Quadrate-Laterosphenoid contact: absent (0); or, present (1).

Character 100. (NEW) Basioccipital recesses: absent (0); or, present as paired foramina located in a median deep depression on the ventral surface of the bone (1).

Character 101. (NEW) Length of the posterodorsal process of the postorbital: short, not reaching the midlength of the supratemporal fenestra (0); or, long, exceeding the midlength of the supratemporal fenestra (1).

Character 102. (NEW) Quadrate fenestra: with participation of the quadratojugal (0); or, exclusively bounded by the quadrate (1).

Character 103. (Clark, 1994) Number of quadrate fenestrae: one (0), or more than one (1).

Character 104. (NEW) Prootic: exposed in dorsal view, on the supratemporal fossa (0); or not exposed in dorsal view (1).

Character 105. (NEW) Exit of the cranial nerves IX-XI: exit the braincase ventromedially (0); or, through a common foramen on the ventromedial region of the paroccipital process (vagus foramen) (1).

Character 106. (NEW) Postzygodiapophyseal laminae on the posterior cervical and anterior dorsal vertebrae: absent or low (0); or, present as sharp lamina delimiting a pit posterior to them on the neural arch (1).

Character 107. (NEW) Length of the radius: shorter than the humerus (0); or, longer than the humerus (1).

Character 108. (NEW)Proximomedial process of the radiale: absent (0); or, present (1)

Character 109. (modified from Clark, 1986; Sereno & Wild, 1992; Nesbitt, 2011) Anterior process of the squamosal: elongated, less than one third of the lateromedial width of the supratemporal fossa (0); transversely broad, more than one third the width of the supratemporal fossa (1); or, very broad, as wide as the width of the supratemporal fossa.

Character 110. (modified from Nesbitt, 2011) Lateral extent of the paroccipital processes: ends lateral to the lateral border of the supratemporal fenestra (including the fossa) (0); or, ends medial to or at the margin of the border of the supratemporal fenestra (1).

Character 111. (modified from Nesbitt, 2011) Anterior end of the dentaries: tapering to a point (0); or, dorsally expanded, forming a distinct step (1).

Character 112. (Parrish, 1994) Anterior part of the dentary: bears teeth (0); or, edentulous (1).

Character 113. (modified from Nesbitt, 2011) Acromial process of scapula: in the same plane of the proximal surface of the scapula (0); or, distinctly raised, forming an abrupt step between the scapular blade and the proximal end of the scapula (1).

Character 114. (Nesbitt, 2011) Coracoid posteroventral edge: smooth (0); or, with a groove (1).

Character 115. (NEW) Scapular contribution to the glenoid: lesser than the coracoid contribution (0); or, equal or greater than the coracoid contribution (1).

Character 116. (Nesbitt, 2011) Humeral proximal head: confined to the proximal surface (0); or, posteriorly expanded and hooked (1).

Character 117. (modified from Wu, Sues & Dong, 1997) Olecranon process on the ulna: present (0); or, absent or very low (1).

Character 118. (NEW) Proximolateral process of the ulna: located at the midpoint of the proximolateral surface of the ulna (0); or, anteriorly displaced, at the level of the anterior process of the ulna (1).

Character 119. (Nesbitt, 2011) Distal end of the ulna: anteroposteriorly compressed or rounded (0); or, with anterior expansion (1).

Character 120. (modified from Clark, Sues & Berman, 2000) Metacarpals II-V configuration: spreading (0); or, compact (1).

Character 121. (modified from Nesbitt, 2011) Dorsoventrally oriented crest dorsal to the supraacetabular crest: absent (0), or, present (1).

Character 122. (modified from Galton, 1976; Clark, 1986; among others) Preacetabular process of the ilium: short and does not extend anteriorly to the acetabulum (0); or, elongated, but shorter than the postacetabular process (1).

Character 123. (Benton & Clark, 1988) Ilium orientation: mainly vertical orientation (0º-20º) (0); or, ventrolaterally deflected about 45º (1).

Character 124. (modified from Nesbitt, 2011) Ventral margin of the acetabulum: convex (0); or, concave (1).

Character 125. (Nesbitt, 2011) Ilium dorsal margin dorsal to the supraacetabular rim: rounded or sharp (0); or, flat (1).

Character 126. (modified form Sereno & Wild, 1992) Obturator foramen on the pubis: present (0); or, absent (1).

Character 127. (modified from Nesbitt, 2011) Ischium medial contact with its antimere: all along its medial margin, but excluding the proximal end (0); or, restricted to the medial edge of the distal part (1).

Character 128. (Nesbitt, 2011) Ischium, distal end: plate-like (0); or, rounded (1).

Character 129. (Nesbitt, 2011) Femur, proximal condylar fold: absent (0); or, present (1).

Character 130. (Nesbitt, 2011) Fibula, proximal end: rounded or elliptical (0); or, mediolaterally compressed (1).

Character 131. (Parrish, 1993) Pedal digit IV, number of phalanges: five (0); or, four (1).

Character 132. (modified from Gauthier, 1986; Parrish, 1993)Pedal digit V, number of phalanges: one or more (0); or, none (1).

Character 133. (modified from Clark, 1994) Ventrally opened notch on ventral edge of rostrum at premaxilla-maxilla contact: absent (0), present as a notch (1), or present as a large notch (2), or present as a notch that is closed ventrally (or largely constrained at its ventral edge) (3).

Character 134. (Nesbitt, 2011) Nasals-frontals contact: transverse (0); or, frontals taper to a point (1).

Character 135. (Weinbaum & Hugerbühler, 2007) Ectopterygoid: single head (0); or, double headed (1).

Character 136. (Benton & Clark, 1988) Ectepicondylar groove: present (0); or, absent (1) (Benton & Clark, 1988). This character has been used in several archosaur studies.

Character 137. (modified from Dyrmala & Zanno, 2016) Antorbital fossa: (0) well defined anterodorsally, yet not well defined along entire length of posterior process of maxilla; (1) well defined, forming complete circumference within the antorbital fenestra.

Character 138. (modified from Dyrmala & Zanno, 2016) Radius, proximal end, medial process: (0) absent, giving the radius a symmetrical aspect in anterior view; (1) present, giving the radius an asymmetrical aspect in anterior view.

**Data matrix**

The following data matrix was used for the phylogenetic analysis. Character scorings between brackets represent polymorphic or uncertain scorings. The latter type of scorings are assigned when there is uncertainty on the assignment of the character state for a given taxon but certain states can be ruled out for that taxon.

*Stagonolepis*

00000000000001000010000000000000000000000?00?000?000?00?00??000?000?00?0??00?0????0??00?00??11??0?000??00000000110000000001000100000000000

*Gracilisuchus*

?1?10??0000101010000???0??000?00?10000000000?000?001???00000000000000000??0000??01010?00?0100?000?000??000??0000?00?????0000001000?0000?0?

*Saurosuchus*

01000?10001000000000000000?0????01000?0001???000000????10000?0000000?0?0100000?0010????10??00?000?000??001??0000????????1010000110?0000?0?

*Postosuchus*

01000??0001100001100000000000000??000000011000001?00?00?00000000000???001?00?0????0??001??10020?0?000??0010000100101001010100?0111000110?0

*Erpetosuchus*

1110???1001100?2011?????000000??0??000?00?0??000?000?00?00000?0?000000?0??00?00??1??0?0?????????0???0??00?0?0?00??0?????????????????0???1?

*Redondavenator*

0?1?????????????????????????0???????????????0??????0???10?00?0??????????????????????????????????????????????????????????????????????0?????

*Carnufex*

01?1??????????????????????01????????00?????10???0??????1??00?00?????????????????????0????????????????????1??????????????????????????0??00?

CM_73372

?????????????????????????????000??????????????0???????0???????????????????????????????0?0?10???????????????0???????????011100?011100??????

*Trialestes*

01?1??????????????????????0??1??????0???????0?????000?0?????000?????????1????????1??0?1???????????????????11??101???1?11???????????????1??

*Litargosuchus*

00?1??1101?00??1100????0??013?????????000??0???????????10?000?0000????????00??????0?0???????????1?0?0??000112100?0?1??????????10?1?1?1?100

*Pseudhesperosuchus*

01??11?101110??0?10????0000?1?10???0??0001?0??000?000?0?0?00000?00?????0??00?0????????1???1001??100????00??111001111111??????????????1?1?0

*Hesperosuchus*

00?1??11111101?0?10????00111112?1100000001000000?0000?0100000?0000??0?????00?00????00?1???10010??0011??001[0 1]11110011101111??010??11?0311111

*Saltoposuchus*

00?1??1101110??0?10??????????12011??00?00100000??0????01???000??0??????0??00??????11??1??01010????0?0????00?1???????1??1?????0?011???1??00

*Terrestrisuchus*

?111??1101100??0?0011??0??011?21???000000100020000000?0?0?000000000000001?0000??0101?01?0??1?00?1001000000?120001111011?110110001110?1?100

*Kayentasuchus*

0010????11111?1?100111????????1?11?0??0?01?0?0???0?????10?0000?0000??????????0????1???1??0011???100????0????[1 2]000??????????1?????1???3???1?

*Dromicosuchus*

00?1???111110??0?10????0??111?1011??00000??00000?000???10000000000?????0??00?00????0001??011110???0?[0 1]??000111000?1?10?1?11101????1???1?110

*Sphenosuchus*

00111011111101121111111001012?????00000001000000?000???10?00?00000000000100000?00?0???1????????010?11??00???1000111101???????????????1110?

*Dibothrosuchus*

00111101111?011211111100011121??0000000111000[1 2]00000000010000000000000000100000000100001?0?????0010010?00001110001011011101011???????31?100

*Junggarsuchus*

00?110?111110?121112???00?0121????11110111111111011001110?00000000000??0?11???0?0100002????????0100011100001100010111111????????????01?100

*Almadasuchus*

0??????10111011201111??01?0???2?1?11??0111???2???0?????????????2010??????11121000?????2??10101?0111111001??110??????????????????1?????????

*Macelognathus*

?1????010?????1??1??112????1??21??1?????????11000??????????????00???????????2??001???????10112??11?1??00??????01????????0??11???11????????

*Hallopus*

?????????????????????????????121??????????????0???0???0???????????????????????????????20?1011[1 2]????????????01????1???0?11?1?????011?1??????

*Hemiprotosuchus*

11111??101101012101????11?1??????1111000??0001???0?????00000001201???100??0000??01000?????????0?1?10011?????2?00????????????????????3???0?

*Protosuchus*

111110?101101012101212211?113?101111100010000100?010?0000000001201000100?20000?1010000100?1001011110011110012000101?1110010111101111311100

*Gobiosuchus*

111111?1011?1012101212011?013?1100110000?0000100?0?????000010111010001000?0?20?1010000?1?01???111110011110?1??00??1?????????????????10??0?

*Orthosuchus*

10111??1011010121012???11???30101111000010?00100?01????00001001201000?00120000?1020??0101?10020111100??11001[1 2]00010111??00101111011?1311110

*Zosuchus*

11111??101101012?012???11?0???????110000100?[0 1]1?????????101010112112101011?01?0110100??1????????1111001111????1?0????????????????????2?????

*Sichuanosuchus*

10111??1011010?21012???11?013?????11000?1000010??01????10?01011[1 2]?1110?01?201?0010100??11??????01?1100??11001100010??1??0????????????21??0?

*Hsisosuchus*

10111??1011010?21012????1?0?????0001000110001100?01?????0??10?1201210?01020120010111001???????01111001?11?011??01010111??????????????1?10?

*Simosuchus*

1111110111101012101212011?013???0001000110010000001???0100030111112101011201200102111[0 1]11?0?0??1111100??11001110010101110?????11?11?100?100

*Notosuchus*

1011110111101012101212011?013?100001000110010100001???0101010111112111010201200102111[0 1]11100002011110011110012000?01011100??10???1???01?110

*Baurusuchus*

1?1?11?1011010121012?2011?00301000010001100111000?1???0101000131112111010201200102111[0 1]1110001?01111001?110011100?01011??00?10110111131?10?

*Dakosaurus*

111011?1001000?21012???11?00???????10000000000???1?????11121012010210101??00100??1?1??1??????????1?01???1???1100????????????????????01?1??

*Metriorhynchus*

101011?100100012?01????11?00??????01000000000000?1?????111[1 2]2012010210101?200100101212?111???????11001??0100?1100???01????001?1??????0??1??

*Cricosaurus*

101011?1001000121112???11?00??0???01000000000000?1?????11022012010210101?200100?01212?1??????????1001??01???1100???01???????????????01?1?0

*Goniopholis*

1?1?1101011010?2101212011?013?10110100001000000??01????111231132112101010201201110[1 2]12?1??0?0??0111100??110011000101011??????????????11????

*Gavialis*

1?1?110101101012101212011?0030100001000010010011100100?1112301321121011102012011102?211110?0??1111100??110011000101011???0???1???1??01?1?0

*Crocodylus*

1?1?110101101012101212011?0130100001000010011011100100?1[0 1]123113211210111020120111001211110?0??1111100??110011000101011?0?00101???1??11?1?0

*Alligator*

1?1?110101101012101212011?0130100001000010011011100100?1002311321121011102012011100?211110?0111111100??1100120001010111000010110111101?1?0

**Strict consensus**

Node numbers are provided to follow the synapomorphies on the synapomorphy list.

**
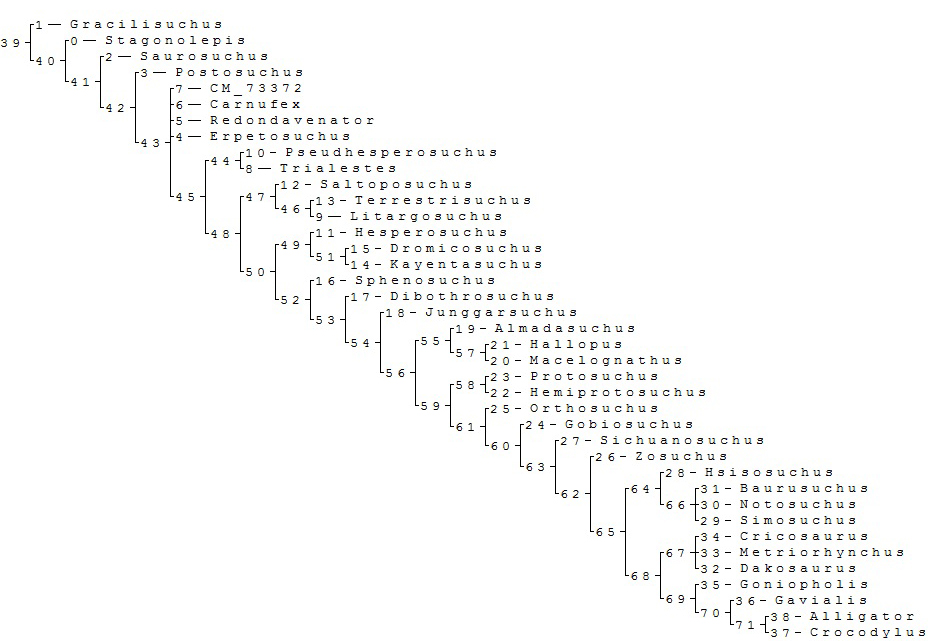
**

**Figure 2S-** Strict consensus of 10 MTPs (L= 348).

**Nodal Support**

The nodal support was evaluated using Bremer support (Bremer, 1994) and two measures based on resampling of characters, bootstrap (Felsenstein, 1985) and jackknife (Farris et al., 1996). Bremer supports were calculated manually, saving all suboptimal trees and evaluating the resulting strict consensus. It is important to note that the Bremer supports command included in TNT showed artificial higher supports, thus we encourage the readers to calculate their Bremer supports manually.The bootstrap and jackknife analyses were conducted performing 10000 psuedoreplicates and a heuristic tree search similar to the one described for the parsimony analysis. Trees obtained during this procedure were summarized with absolute and GC frequencies (Goloboff et al., 2003). The consensus trees depicted are reduced consensus, as for nodal support calculations the conflicting taxa (*Redondavenator, Carnufex, Trialestes,* and *Litargosuchus*), previosly identified were excluded from it.

**
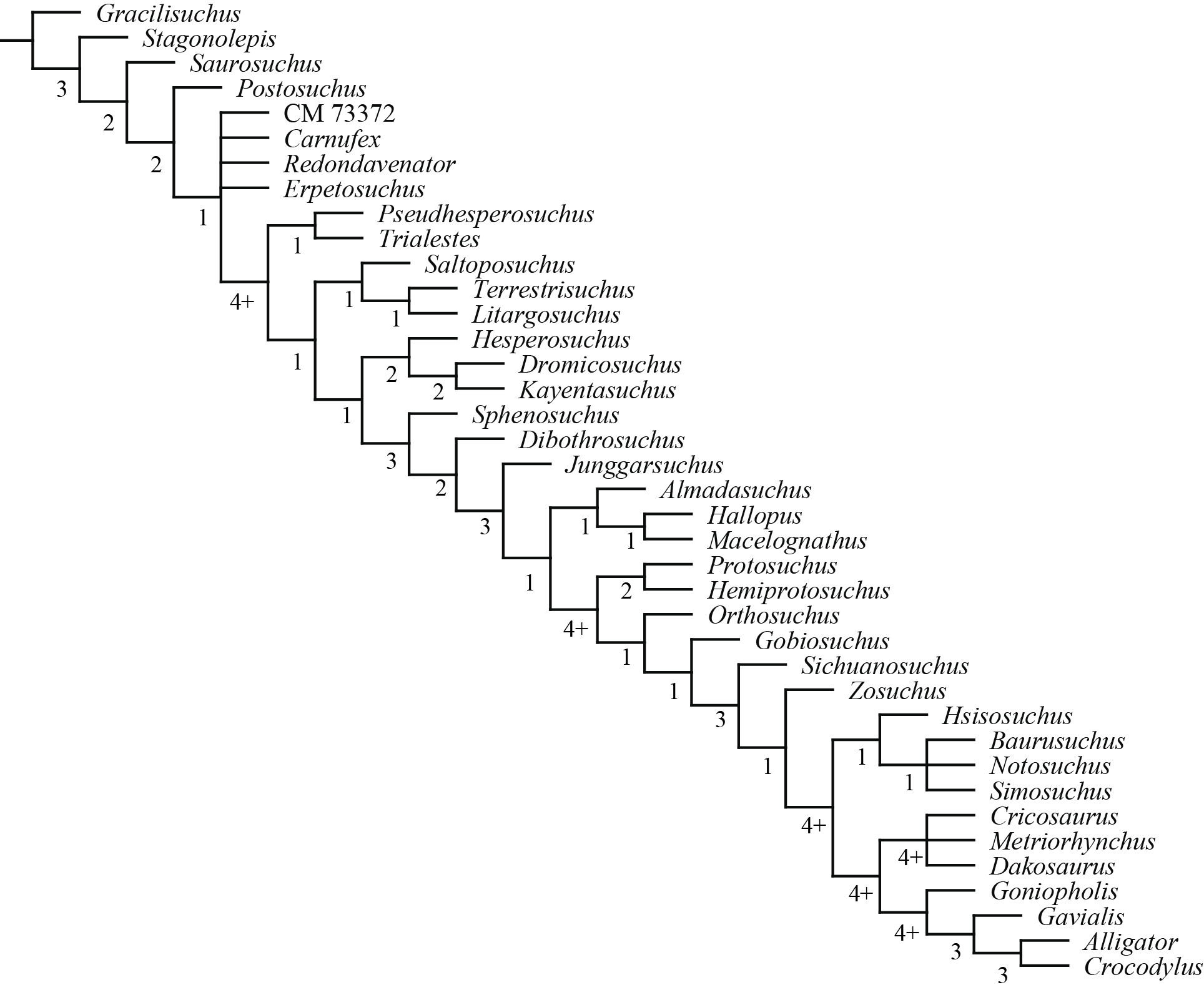
**

**Figure 3S-** Consensus depicting the Bremer support values.

**Figure 4S-** Reduced consensus depicting the absolute Bootstrap support values.

**Figure 5S-** Reduced consensus depicting the GC Bootstrap support values.

**Figure 6S-** Reduced consensus depicting the Jacknife support values.

**Figure 7S-** Reduced consensus depicting the GC Jacknife support values.

**
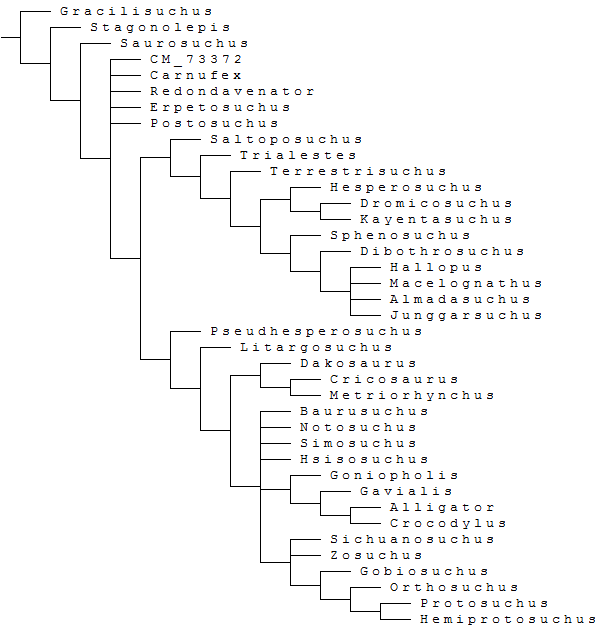
**

**Figure 8S-** Strict consensus of 369 MTPs under the constrain where the thalattosuchians are forced to be the sister group of Crocodyliformes (L = 361).

**List of synapomorphies**

The following list is the complete set of unambiguous synapomorphies of the 10 MPTs obtained in the phylogenetic analysis conducted in TNT. It should be noted that the TNT program starts numbering the characters from 0 instead of 1. Node numbers refer to the enumeration of nodes of the strict consensus shown above.

*Stagonolepis* :

All trees:

Char. 1: 1 --> 0

Char. 18: 0 --> 1

Char. 33: 1 --> 0

Char. 92: 0 --> 1

Char. 111: 0 --> 1

*Gracilisuchus* :

All trees:

No autapomorphies:

*Saurosuchus* :

All trees:

No autapomorphies:

*Postosuchus* :

All trees:

Char. 42: 0 --> 1

Char. 48: 0 --> 1

Char. 93: 1 --> 2

Char. 110: 0 --> 1

Char. 112: 1 --> 0

*Erpetosuchus* :

Some trees:

Char. 0: 0 --> 1

Char. 15: 0 --> 2

Char. 18: 0 --> 1

Char. 136: 0 --> 1

*Redondavenator* :

All trees:

No autapomorphies:

*Carnufex* :

All trees:

Char. 43: 0 --> 1

CM_73372 :

All trees:

No autapomorphies:

*Trialestes* :

All trees:

Char. 110: 0 --> 1

*Litargosuchus* :

All trees:

Char. 15: 0 --> 1

Char. 28: 1 --> 3

Char. 109: 0 --> 1

Char. 113: 1 --> 0

Char. 131: 0 --> 1

*Pseudhesperosuchus* :

All trees:

No autapomorphies:

*Hesperosuchus* :

All trees:

Char. 105: 0 --> 1

Char. 109: 0 --> 1

Char. 110: 0 --> 1

Char. 137: 0 --> 1

*Saltoposuchus* :

All trees:

Char. 82: 0 --> 1

Char. 106: 1 --> 0

Char. 116: 0 --> 1

*Terrestrisuchus* :

All trees:

Char. 1: 0 --> 1

*Kayentasuchus* :

All trees:

Char. 3: 1 --> 0

Char. 12: 0 --> 1

Char. 17: 1 --> 0

Char. 90: 1 --> 0

*Dromicosuchus* :

All trees:

No autapomorphies:

*Sphenosuchus* :

All trees:

No autapomorphies:

*Dibothrosuchus* :

All trees:

Char. 5: 0 --> 1

Char. 26: 0 --> 1

Char. 32: 1 --> 0

Char. 33: 1 --> 0

Char. 100: 1 --> 0

*Junggarsuchus* :

All trees:

Char. 37: 0 --> 1

Char. 42: 0 --> 1

Char. 43: 0 --> 1

Char. 46: 0 --> 1

Char. 47: 0 --> 1

Char. 49: 0 --> 1

Char. 53: 0 --> 1

Char. 54: 0 --> 1

Char. 132: 3 --> 0

*Almadasuchus* :

All trees:

Char. 45: 1 --> 2

*Macelognathus :*

All trees:

No autapomorphies:

*Hallopus* :

All trees:

No autapomorphies:

*Hemiprotosuchus* :

All trees:

No autapomorphies:

*Protosuchus* :

All trees:

No autapomorphies:

*Gobiosuchus* :

All trees:

Char. 31: 0 --> 1

Char. 63: 2 --> 1

Char. 94: 0 --> 1

Char. 133: 1 --> 0

*Orthosuchus* :

All trees:

Char. 81: 1 --> 2

Char. 136: 0 --> 1

*Zosuchus* :

All trees:

Char. 78: 0 --> 1

*Sichuanosuchus* :

All trees:

No autapomorphies:

*Hsisosuchus* :

All trees:

Char. 64: 1 --> 0

Some trees:

Char. 44: 0 --> 1

*Simosuchus* :

All trees:

Char. 1: 0 --> 1

Char. 45: 1 --> 0

Char. 57: 1 --> 0

Char. 59: 1 --> 3

Char. 72: 0 --> 1

Char. 94: 0 --> 1

Char. 133: 1 --> 0

*Notosuchus* :

All trees:

Char. 108: 1 --> 2

Char. 109: 1 --> 0

Char. 136: 0 --> 1

*Baurusuchus* :

All trees:

Char. 27: 1 --> 0

Char. 59: 1 --> 0

Char. 62: 1 --> 3

Char. 132: 0 --> 3

Some trees:

Char. 44: 0 --> 1

*Dakosaurus* :

All trees:

Char. 1: 0 --> 1

Char. 59: 2 --> 1

*Metriorhynchus* :

All trees:

No autapomorphies:

*Cricosaurus* :

All trees:

Char. 17: 0 --> 1

Char. 57: 1 --> 0

*Goniopholis* :

All trees:

Char. 32: 0 --> 1

Char. 33: 0 --> 1

Char. 132: 0 --> 1

*Gavialis* :

All trees:

Char. 27: 1 --> 0

*Crocodylus* :

All trees:

Char. 132: 0 --> 1

*Alligator* :

All trees:

Char. 57: 1 --> 0

Char. 108: 1 --> 2

Node 40 :

All trees:

No synapomorphies

Node 41 :

All trees:

Char. 10: 0 --> 1

Char. 13: 1 --> 0

Char. 105: 0 --> 1

Char. 120: 0 --> 1

Char. 126: 1 --> 0

Char. 127: 0 --> 1

Char. 128: 0 --> 1

Node 42 :

All trees:

Char. 17: 0 --> 1

Char. 129: 0 --> 1

Char. 133: 0 --> 1

Char. 134: 0 --> 1

Node 43 :

Some trees:

Char. 2: 0 --> 1

Char. 7: 0 --> 1

Char. 121: 0 --> 1

Node 44 :

All trees:

Char. 116: 0 --> 1

Node 45 :

Some trees:

Char. 28: 0 --> 1

Char. 30: 0 --> 1

Char. 86: 0 --> 1

Char. 107: 0 --> 1

Char. 119: 0 --> 1

Char. 135: 0 --> 1

Node 46 :

All trees:

Char. 11: 1 --> 0

Char. 17: 1 --> 0

Char. 108: 1 --> 2

Node 47 :

All trees:

Char. 93: 1 --> 0

Node 48 :

All trees:

Char. 1: 1 --> 0

Node 49 :

All trees:

Char. 26: 0 --> 1

Char. 136: 0 --> 1

Node 50 :

All trees:

Char. 8: 0 --> 1

Char. 83: 1 --> 0

Char. 100: 0 --> 1

Node 51 :

All trees:

Char. 91: 0 --> 1

Char. 92: 0 --> 1

Node 52 :

All trees:

Char. 15: 0 --> 2

Char. 18: 0 --> 1

Char. 28: 1 --> 2

Node 53 :

All trees:

Char. 6: 1 --> 0

Char. 39: 0 --> 1

Char. 40: 0 --> 1

Char. 45: 0 --> 1

Char. 113: 1 --> 0

Node 54 :

All trees:

Char. 34: 0 --> 1

Char. 35: 0 --> 1

Char. 73: 0 --> 1

Char. 106: 1 --> 0

Node 55 :

All trees:

Char. 76: 0 --> 2

Char. 89: 0 --> 1

Char. 90: 1 --> 0

Char. 91: 0 --> 1

Node 56 :

All trees:

Char. 8: 1 --> 0

Char. 24: 0 --> 1

Char. 65: 0 --> 1

Char. 97: 0 --> 1

Char. 98: 0 --> 1

Char. 104: 0 --> 1

Node 57 :

All trees:

Char. 92: 0 --> 1

Node 58 :

All trees:

Char. 26: 0 --> 1

Char. 108: 1 --> 2

Node 59 :

All trees:

Char. 0: 0 --> 1

Char. 11: 1 --> 0

Char. 12: 0 --> 1

Char. 13: 1 --> 0

Char. 17: 1 --> 0

Char. 21: 1 --> 2

Char. 23: 0 --> 1

Char. 39: 1 --> 0

Char. 41: 1 --> 0

Char. 73: 1 --> 2

Char. 79: 0 --> 1

Char. 95: 0 --> 1

Char. 100: 1 --> 0

Char. 103: 0 --> 1

Char. 119: 1 --> 0

Node 60 :

All trees:

Char. 32: 1 --> 0

Char. 33: 1 --> 0

Char. 61: 0 --> 1

Char. 76: 0 --> 2

Char. 87: 0 --> 1

Node 61 :

All trees:

Char. 59: 0 --> 1

Char. 88: 0 --> 1

Node 62 :

All trees:

Char. 66: 1 --> 2

Char. 109: 0 --> 1

Node 63 :

All trees:

Char. 55: 0 --> 1

Char. 66: 0 --> 1

Char. 67: 0 --> 1

Char. 71: 0 --> 1

Node 64 :

All trees:

Char. 39: 0 --> 1

Node 65 :

All trees:

Char. 34: 1 --> 0

Char. 82: 0 --> 1

Char. 83: 0 --> 1

Char. 132: 2 --> 0

Node 66 :

All trees:

Char. 43: 0 --> 1

Char. 63: 2 --> 1

Char. 81: 1 --> 2

Char. 84: 0 --> 1

Node 67 :

All trees:

Char. 9: 1 --> 0

Char. 12: 1 --> 0

Char. 27: 1 --> 0

Char. 40: 1 --> 0

Char. 49: 0 --> 1

Char. 63: 2 --> 0

Char. 65: 1 --> 0

Char. 75: 1 --> 0

Char. 76: 2 --> 1

Char. 98: 1 --> 0

Char. 100: 0 --> 1

Char. 103: 1 --> 0

Node 68 :

All trees:

Char. 45: 1 --> 0

Char. 56: 0 --> 1

Char. 58: 0 --> 2

Char. 59: 1 --> 2

Char. 62: 1 --> 2

Char. 84: 0 --> 2

Node 69 :

All trees:

Char. 59: 2 --> 3

Char. 62: 2 --> 3

Char. 78: 0 --> 1

Char. 80: 0 --> 1

Char. 81: 1 --> 0

Char. 109: 1 --> 0

Node 70 :

All trees:

Char. 43: 0 --> 1

Char. 46: 0 --> 1

Char. 50: 1 --> 0

Char. 70: 0 --> 1

Char. 94: 0 --> 1

Node 71 :

All trees:

Char. 44: 0 --> 1

Char. 82: 12 --> 0

**Supplementary Literature Cited**

Benton MJ, Clark JM. 1988. Archosaur phylogeny and the relationships of the Crocodylia. In: Benton MJ, ed. *The Phylogeny and Classification of Tetrapods, Volume 1: Amphibians, Reptiles, Birds.* Oxford: Clarendon Press, 295-338.

Benton MJ, Walker AD. 2002. *Erpetosuchus*, a crocodile-like basal archosaur from the Late triassic of Elgin, Scotland. Zoological Journal of the Linnean Society 136 :25-47.

Blaniville, H. M. D. de. 1853. Lettre sur les Crocodiliens vivants et fossils. Memoires de la Société Linneenne de Normandie, Caen 9:109-120.

Bonaparte, J. F. 1972. Los tetrápodos del sector superior de la formación Los Colorados, La Rioja, Argentina. Opera Lilloana 22:1-183.

Bremer, K. 1994. Branch support and tree stability. Cladistics 10:295-304.

Brochu, C. A. 1999.Phylogenetics, taxonomy, and historical biogeography of Alligatoroidea.Memoir of the Society of Vertebrate Paleontology 4:1-92.

Buckley, G. A., C. A. Brochu, D. W. Krause, and D. Pol. 2000.A pug-nosed crocodyliform from the Late Cretaceous of Madagascar.Nature 405:941-944.

Bonaparte JF. 1972. Los tetrápodos del sector superior de la Formación Los Colorados, La Rioja, Argentina (Triásico Superior). 1 Parte. Opera Lilloana 22:1-183.

Busbey ABIII, Gow C. 1984. A new protosuchian crocodile from the Upper Triassic Elliot Formation of South Africa. Palaeontologia Africana 25:127-149.

Carvalho, I. S., A. C. A. Campos, and P. H. Nobre. 2005. *Baurusuchus salgadoensis*, a new Crocodylomorpha from the Bauru Basin (Cretaceous), Brazil. Gondwana Research 8:11-30.

Clark, J. M. 1986.Phylogenetic relationships of the crocodylomorph Archosaurs.Ph.D.dissertation, University of Chicago, 556 pp.

Clark, J. M. 1994. Patterns of evolution in Mesozoic Crocodyliformes; pp. 84-97 in N. C. Fraser and H.-D. Sues (eds.), In the shadow of dinosaurs. Cambridge University Press, Cambridge.

Clark JM, Sues H-D, Berman DS. 2000. A new specimen of *Hesperosuchus agilis* from the Upper Triassic of New Mexico and the interrelationships of basal crocodylomorph archosaurs. Journal of Vertebrate Paleontology 20:683-704.

Clark JM, Sues H-D. 2002. Two new basal crocodylomorph archosaurs from the Lower Jurassic and the monophyly of the Sphenosuchia. Zoological Journal of the Linnean Society 136:77-95.

Clark JM, Xu X, Forster CA, Wang Y. 2004. A Middle Jurassic “sphenosuchian” from China and the origin of the crocodilian skull. Nature 430:1021-1024.

Chaterjee S. 1985. *Postosuchus*, a new thecodontian reptile from the Triassic of Texas and the origin of Tyrannosaurs. Philosophical Transactions of the Royal Society of London B 309:395-460.

Colbert, E. C. 1946.Sebecus, representative of a peculiar suborder of fossil Crocodilia from Patagonia.Bulletin of the American Museum of Natural History 87:221-270.

Colbert EC. 1952. A pseudosuchian reptile from Arizona. Bulletin of the American Museum of Natural History 99:561-592.

Colbert, E. C., and C. C. Mook. 1951. The ancestral crocodile *Protosuchus*. Bulletin of the American Museum of Natural History 97:143-182.

Crush, P. J. 1984. A late Upper Triassic sphenosuchid crocodilian from Wales.Palaeontology 27:131-157.

Dyrmala SA, Zanno LE. 2016. Osteology of *Carnufex carolinensis* (Archosauria: Pseudosuchia) from the Pekin Formation of North Carolina and its implications for Early Crocodylomorph Evolution. PLoS ONE 11 (6):e0157528. Doi: 10.137/journal.pone.0157528.

Eudes-Deslongchamps, E. 1863. Memoires sur les téléosauriens de l'epoque Jurassique du département du Calvados. Memoires de la Société Linneenne de Normandie 13:1-138.

Farris, J. S., V. A. Albert, M. Källersjö, D. Lipscomb, and A. C. Kludge. 1996. Parsimony jackknifing outperforms neighbor-joining. Cladistics 12:99-124.

Felsenstein, J. 1985. Confidence limits on phylogenies: an approach using the bootstrap. Evolution 39:783-791.

Galton, P.M. 1976. Prosauropod dinosaurs (Reptilia: Saurischia) of North America. Postilla 169:1-98.

Gasparini, Z. 1971. Los Notosuchia del Cretácico de América del Sur como un nuevo infraorden de los Mesosuchia (Crocodilia). Ameghiniana 8:83-103

Gasparini, Z., and D. Dellapé. 1976. Un nuevo cocodrilo marino (Thalattosuchia, Metriorhynchidae) de la Formación Vaca Muerta (Jurásico, Tithoniano) de la Provincia de Neuquén. Actas I Congreso Geológico Chileno, Santiago 1976:C1-C21.

Gauthier, J.A. 1986. Saurischian monophyly and the origin of birds. Memoirs of the California Academy of Science 8: 1-55.

Georgi JA, Krause DW. 2010. Postcranial axial skeleton of *Simosuchus* *clarki* (Crocodyliformes: Notosuchia) from the Late Cretaceous of Madagascar. Journal of Vertebrate Paleontology 30(s1):99-121.

Göhlich UB, Chiappe LM, Clark JM, Sues H-D. 2005. The systematic position of the Late Jurassic alleged dinosaur *Macelognathus* (Crocodylomorpha: Sphenosuchia). Canadian Journal of Earth Sciences 42:307-321. DOI: 10.1139/E05-005.

Goloboff, P. A., J. S. Farris, M. Källersjö, B. Oxelman, M. J. Ramirez, and C. A. Szumik. 2003. Improvements to resampling measures of group support. Cladistics 19:324-332.

Krause, D. W., and N. J. Kley. 2010. *Simosuchus clarki* (Crocodyliformes: Notosuchia) from the Late Cretaceous of Madagascar. Memoirs of the Society of Vertebrate Paleontology 10:1-236.

Long RA, Murry PA. 1995. Late Triassic (Carnian and Norian) tetrapods from the southwestern United States. New Mexico Museum of Natural History and Science 4: 1-254.

Marsh OC. 1884. A new order of extinct Jurassic reptile (Macelognatha). American Journal of Science 27(3):341.

Mook, C. C.1942.Skull characters of *Amphicotylus lucasii* Cope.American Museum Novitates 1202:1-5.

Nascimento, P. M., and H. Zaher. 2010. A new species of *Baurusuchus* (Crocodyliformes, Mesoeucrocodylia) from the Upper Cretaceous of Brazil, with the first complete postcranial skeleton described for the family Baurusuchidae. Papéis Avulsos de Zoologia 50:323-361.

Nash, D. S. 1975. The morphology and relationships of a crocodilian, *Orthosuchus stormbergi*, from the Upper Triassic of Lesotho.Annals of the South African Museum 67:227-329.

Nesbitt SJ. 2011. The early evolution of archosaurs: relationships and the origin of major clades. Bulletin of the American Museum of Natural History 352:1-292.

Nesbitt SJ, Irmis RB, Lucas SG, Hunt AP. 2005. A giant crocodylomorph from the Upper Triassic of New Mexico. Paläontologische Zeitschrift 79/4:471-478.

Ortega, F., Z. Gasparini, A. D. Buscalioni, and J. O. Calvo. 2000. A new species of *Araripesuchus* (Crocodylomorpha, Mesoeucrocodylia) from the Lower Cretaceous of Patagonia (Argentina). Journal of Vertebrate Paleontology 20:57-76.

Osmólska, H. 1972. Preliminary note on a crocodilian from the Upper Cretaceous of Mongolia.Palaeontologia Polonica 27:43-47.

Osmólska, H., S. Hua, and E. Buffetaut.1997.*Gobiosuchus kielanae* (Protosuchia) from the Late Cretaceous of Mongolia: anatomy and relationships. Acta Paleontologica Polonica 42:257-289.

Ostrom JH. 1971. On the systematic position of *Macelognathus vagans*. Postilla 153:1-10.

Parrish JM. 1993. Phylogeny of the Crocodyotarsi, with reference to archosaurian and crurotarsan monophyly. Journal of Vertebrate Paleontology 13:287-308.

Parrish JM. 1994. Cranial osteology of *Longosuchus* *meadei* and the phylogeny and distribution of the Aetosauria. Journal of Vertebrate Paleontology 14(2):196-209.

Peng, G.-Z., and Shu, C.-K. 2005. A new species of *Hsisosuchus* from the Late Jurassic of Zigong, Sichuan, China.Vertebrata Palasiatica 43:312-324.

Peyer K, Carter JG, Sues H-D, Novak SE, Olsen PE. 2008. A new suchian archosaur from the Upper Triassic of North Carolina. Journal of Vertebrate Paleontology 28:363-381.

Pol D, Rauhut OWM, Lecuona A, Leardi JM, Xu X, Clark JM. 2013. A new fossil from the Jurassic of Patagonia reveals the early basicranial evolution and the origins of Crocodyliformes. Biological Reviews 88:862-872. DOI: 10.1111/brv.12030.

Pol, D., and M. A. Norell.2004a.A new crocodyliform from Zos Canyon Mongolia.American Museum Novitates 3445:1-36.

Pol, D., A. H. Turner, and M. A. Norell. 2009. Morphology of the Late Cretaceous crocodylomorph *Shamosuchus djadochtaensis* and a discussion of neosuchian phylogeny as related to the origin of Eusuchia. Bulletin of American Museum of Natural History 324:1-103.

Reig OA. 1963. La presencia de dinosaurios saurisquios en los “estratos de Ischigualasto” (Mesotriásico Superior) de las provincias de San Juan y La Rioja (República Argentina). Ameghiniana 3:3-20.

Romer, A. S. 1972.The Chañares (Argentina) Triassic reptile fauna. XIII. An early ornithosuchid pseudosuchian, Gracilisuchus stipanicicorum, gen. et sp. nov. Breviora 389:1-24.

Salisbury, S. W., P. M. A. Willis, S. Peitz, and P. M. Sander. 1999. The crocodilian *Goniopholis simus* from the Lower Cretaceous of north-western Germany; pp. 121–148 in D. Unwin (ed.), Cretaceous Fossil Vertebrates. Special Papers in Palaeontology, 60.

Sereno PC, Wild R. 1992. *Procompsognathus*: theropod, “thecodont” or both? Journal of Vertebrate Paleontology 12:435-458.

Sertich, J. J. W., and J. R. Groenke. 2010. Appendicular Skeleton of *Simosuchus clarki* (Crocodyliformes: Notosuchia) from the Late Cretaceous of Madagascar. Society of Vertebrate Paleontology Memoir 10:122-153.

Sues H-D, Olsen PE, Carter JG, Scott DM. 2003. A new crocodylomorph archosaur from the Upper Triassic of North Carolina. Journal of Vertebrate Paleontology 23:329-343.

Vignaud, P., and Z. Gasparini. 1996. New *Dakosaurus* (Crocodylomorpha, Thalattosuchia) in the Upper Jurassic of Argentina. Comptes Rendus de l'Académie de Sciences Paris, Sciences de la Terre 322:245-250.

Walker AD. 1961. Triassic reptiles from the Elgin area: *Stagonolepis*, *Dasygnathus* and their allies. Philosophical Transactions of the Royal Society of London, B 244:103-204.

Walker AD. 1970. A revision of the Jurassic reptile *Hallopus* *victor* (Marsh) with remarks on the classification of the crocodiles. Philosophical Transactions of the Royal Society of London, B 257:323-372.

Walker AD. 1990. A revision of *Sphenosuchus acutus* Haughton, a crocodylomorph reptile from the Elliot Formation (late Triassic or early Jurassic) of South Africa. Philosophical Transactions of the Royal Society of London, B 330:1-120.

Weinbaum JC. 2002. Osteology and relationships of *Postosuchus kirkpatricki* (Archosauria: Crurotarsi). Ms. Thesis, Texas Tech University.

Wenz, S. 1968. Contribution a l´étude du genere Metriorhynchus: crâne et moulage endocranien de *Metriorhynchus superciliosus*. Annals de Paléontologie 54:148-191.

Wu, X.-C., and S. Chatterjee. 1993. *Dibothrosuchus elaphros*, a crocodylomorph from the Lower Jurassic of China and the phylogeny of the Sphenosuchia. Journal of Vertebrate Paleontology 13:58-89.

Wu, X.-C., and H.-D. Sues. 1996. Anatomy and phylogenetic relationships of *Chimaeresuchus paradoxus*, an unusual crocodyliform reptile from the Lower Cretaceous of Hubei, China. Journal of Vertebrate Paleontology 16:688-702.

Wu, X.-C., H.-D. Sues, and Z.-M. Dong. 1997. *Sichuanosuchus shuhanensis*: a new ?Early Cretaceous protosuchian (Archosauria: Crocodyliformes) from Sichuan (China), and the monophyly of Protosuchia. Journal of Vertebrate Paleontology 17:89-103.

Zanno LE, Drymala S, Nesbitt SJ, Schneider VP. 2015. Early crocodylomorph increases top tier predator diversity during rise of dinosaurs. Scientific Reports 5:9276. DOI:10.1038/srep09276 (2015).
